# Infant Gaze Following Is Stable Across Markedly Different Cultures and Resilient to Family Adversities Associated With War and Climate Change

**DOI:** 10.1177/09567976251331042

**Published:** 2025-04-21

**Authors:** Gustaf Gredebäck, Kim Astor, Herbert Ainamani, Linda van den Berg, Linda Forssman, Jonathan Hall, Joshua Juvrud, Ben Kenward, Samson Mhizha, Wangchuk, Pär Nyström

**Affiliations:** 1 Department of Psychology, Uppsala University; 2 Department of Mental Health, Kabale University School of Medicine; 3 Department of Peace and Conflict Research, Uppsala University; 4 Department of Game Design, Uppsala University; 5 School of Psychology, Social Work and Public Health, Oxford Brookes University; 6 Department of Applied Psychology, University of Zimbabwe; 7 Public Heath and Allied Health Sciences Department, Khesar Gyalpo University of Medical Sciences of Bhutan

**Keywords:** infant, eye tracking, social cognition

## Abstract

Gaze following in infancy allows triadic social interactions and a comprehension of other individuals and their surroundings. Despite its importance for early development, its ontology is debated, with theories suggesting that gaze following is either a universal core capacity or an experience-dependent learned behavior. A critical test of these theories among 809 nine-month-olds from Africa (Uganda and Zimbabwe), Europe (Sweden), and Asia (Bhutan) demonstrated that infants follow gaze to a similar degree regardless of environmental factors such as culture, maternal well-being (postpartum depression, well-being), or traumatic family events (related to war and/or climate change). These findings suggest that gaze following may be a universal, experience-expectant process that is resilient to adversity and similar across a wide range of human experiences—a core foundation for social development.

Developing the ability to share attention with others represents an important cornerstone of individual children’s development (supporting language development, emotion regulation, and social understanding; Del Bianco et al., 2019; Lasch et al., 2023) while also providing a foundation for the social transmission of knowledge across generations and ages (Ishikawa & Senju, 2023; Meltzoff, 2007; O’Madagain & Tomasello, 2021). As such, joint attention is a fundamental capacity tightly associated with learning and the development of both individuals and societies.

Despite its importance, little is known about the developmental roots of joint attention, and more specifically, the processes that allow infants to follow others’ gaze (Astor & Gredebäck, 2022). Empiricist theories suggest that gaze following is an experience-dependent process that relies on high-quality social interactions with caregivers (Del Bianco et al., 2019; Silverstein et al., 2021; Triesch et al., 2006). This claim is supported by studies from the global North demonstrating that insecure attachment and maternal depression (Astor et al., 2020) can impact gaze-following abilities. In contrast, nativist theories maintain that the ability to follow gaze develops independently from the quality of social interactions they experience (Del Bianco et al., 2019; Emery, 2000; Spelke, 2022). This claim is supported by the widespread phylogenetic distribution of gaze following across species (Zeiträg et al., 2022) and a study that showed that infants whose parents were blind could still follow gaze (Senju et al., 2015).

A related discussion centers on cultural diversity, or more specifically whether gaze following is a universal phenomenon that unfolds similarly across cultures. Recent cross-cultural work has argued that children develop joint attention in ways that are specific to their cultural contexts, suggesting that gaze following is a phenotype supported by distal child-rearing practices prevalent in the global North (Bard et al., 2021). At the same time, studies documenting gaze following in Asia (Astor et al., 2022; Ishikawa & Itakura, 2019), Europe (Gredebäck et al., 2010; Michel et al., 2021), the United States (Tenenbaum et al., 2015), and Oceania (Hernik & Broesch, 2019) suggest that this ability exists at a basic level in diverse cultural settings. To date, only a single comparative study has assessed infants’ tendencies to follow gaze across cultures, revealing a more pronounced impact of maternal postpartum depression on infants’ gaze following in Sweden than in Bhutan (Astor et al., 2022). The relatively small sample sizes used in these studies represent a limitation that may have led to the conflicting interpretations found in previous research.

This study aimed to assess the stability of gaze following across markedly different cultures and whether maternal mental health alters infants’ tendency to follow gaze. We also sought to determine whether mothers’ experiences of potentially traumatic events associated with war (witnessing a killing, being tortured), climate change (drought), or a combination of factors (severe lack of food and water) influence infants’ tendency to follow gaze. It is well established that poor mental health and/or extreme traumatic events can deplete a mother’s psychological resources, thus impairing both the quality and frequency of interactions between parent and infant (Bjørnebekk et al., 2015; Goodman et al., 2020; Slomian et al., 2019; Zou et al., 2019). With these data, we have an unprecedented opportunity to clarify the ontogenetic origin of gaze following, and by extension joint attention, across the globe.

## Research Transparency Statement

### General disclosures

**Conflicts of interest:** All authors declare no conflicts of interest. **Funding:** The study was funded by Knut and Alice Wallenberg Foundation Wallenberg Academy Fellows Grants KAW 2012.0120 and KAW 2017.0284. **Artificial intelligence:** No AI-assisted technologies were used in this research or the creation of this article. **Ethics:** This research received approval from a local ethics board at all locations.

### Study disclosures

**Preregistration:** No aspect of the study was preregistered. The project is described at https://osf.io/t7rxn/files/osfstorage. **Materials:** All study materials are publicly available (https://osf.io/j69bm/files/osfstorage). **Data:** All primary gaze data and questionnaire primary data used in this publication are publicly available (https://osf.io/dx3zv/files/osfstorage). **Analysis scripts:** All analysis scripts are publicly available (https://osf.io/dx3zv/files/osfstorage). **Computational reproducibility:** Because of resource constraints, the journal’s STAR team did not complete a reproducibility check for this manuscript.

## Method

### Participants

**Fig. 1.**
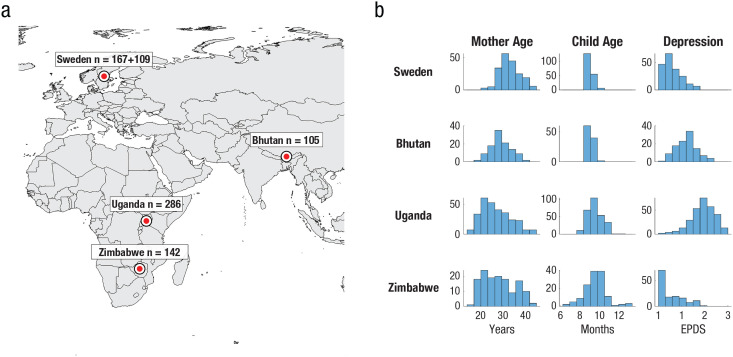
Location of field sites for data collection. The data were collected in (a) Bhutan (Buddhist lower-middle-income country; sample of 120 families from the capital Thimphu), Sweden (Christian high-income country; samples of 118 and 191 families from Uppsala, a university town), Uganda (Christian low-income country; sample of 294 refugee families that live in Nakivale, a refugee camp with refugees from the Democratic Republic of Congo, South Sudan, and Rwanda), and Zimbabwe (Christian lower-middle-income country sample of 147 families that live in high-density areas in the capital, Harare). For more detailed descriptive information, including ethnicities, income, and family characteristics, see Table 1. Data-collection sites were selected to vary environmental factors affecting families. The (b) participant characteristics show variation in mother age in years, infant age in months, and the average score on the Edinburgh Postnatal Depression Scale (EPDS). The mother’s mental health, measured using the EPDS, serves as a proxy for the mother-child interaction and social environment of the infant.

We analyzed data from 809 nine-month-old infants spanning three continents (Asia, Africa, and Europe) and four countries (Sweden, Bhutan, Uganda, and Zimbabwe; Fig. 1). Specifically, the data consisted of five data sets collected at different locations and time points using conceptually the same stimuli at all sites. Three data sets—Sweden Data Set 1 (hereafter Sweden 1), Uganda, and Zimbabwe (*n* = 595)—were novel with identical stimuli, and these data sets have not been analyzed or published previously. Two of these data sets—Sweden Data Set 2 (hereafter Sweden 2) and Bhutan (*n* = 214)—have been previously published (Astor et al., 2022) and were included without reprocessing. Data from 51 additional infants were excluded because of no usable eye-tracking data (*n* = 6) or lack of fixations on either of the two objects in the scene, or missing questionnaire data (*n* = 45: Sweden 1 *n* = 28, Sweden 2 *n* = 1, Bhutan *n* = 1, Uganda *n* = 10, Zimbabwe *n* = 5). The average age of the mothers was 29.2 years (*SD* = 5.9) at the day of data collection, and their average Edinburgh Postnatal Depression Scale (EPDS) score was 1.097 (*SD* = 0.780). The infants’ age was 9.4 months (*SD* = 0.7), and 393 were reported girls, 410 were reported boys, and six infants were missing data. All families participated with informed consent and received a small gift (i.e., a gift card or toy) for participating. Descriptive data of mother and infant ages are found in Table 1. Missing data for child or mother age because of mothers not knowing or data-registration errors were replaced with the mean age at the individuals’ locations (15 mothers, 42 infants). All procedures were performed in accordance with ethical approval in the country in which the data were collected.

**Table 1.** Descriptive Statistics for the Different Data Sets, Individual Countries, and Total Sample

	Sweden 1	Sweden 2	Bhutan	Uganda	Zimbabwe	Total sample
*n*	166	109	105	286	143	809
Mother’s age, years	32.8 (4.3)	30.7 (3.7)	29.1 (4.7)	27.1 (6.4)	28.1 (6.5)	29.2 (5.9)
Child’s age, months	9.9 (0.3)	9.2 (0.3)	9.0 (0.4)	9.6 (0.7)	9.5 (1.1)	9.4 (0.7)
Girls (boys), *n*	84 (82)	56 (53)	49 (56)	138 (145)	66 (74)	393 (410)
Gaze-following difference score	1.4 (2.4)	1.2 (2.1)	1.6 (1.9)	1.2 (2.4)	1.4 (2.4)	1.3 (2.3)
EPDS	0.6 (0.4)	0.5 (0.4)	1.2 (0.4)	1.9 (0.5)	0.5 (0.5)	1.1 (0.8)
Questionnaires	EPDS, WAEC, LS	EPDS	EPDS	EPDS, WAEC, LS	EPDS, WAEC, LS	
Year of data collection	2022	2014–2017	2018	2023	2022–2023	
Location	Uppsala (university town)	Uppsala (university town)	Thimphu (capital)	Nakivale (refugee camp)	Tafara, Harare (high-density area)	
Ethnicity and/or country of birth mother	Country: Swedish (total *n* = 178), other (*n* = 3)	Country: Swedish (total *n* = 111), other (*n* = 7)	Ethnicity: Ngalop (*n* = 29), Sharchop (*n* = 45), Lhotsampa (*n* = 16), other (*n* = 10)	Ethnicity: Hutu (*n* = 131), Tutsi (*n* = 124), other (*n* = 18); mother’s birth country: DRC (*n* = 167), Burundi (*n* = 69), Rwanda (*n* = 36), Tanzania (*n* = 16), other (*n* = 8)	Ethnicity: Shona (*n* = 136), other (*n* = 9)	
Religion	Atheist (*n* = 164), Christian (*n* = 31)		Buddhist (*n* = 92), Hindu (*n* = 14), Christian (*n* = 2)^a^	Christian (*n* = 281), Muslim (*n* = 13)	Christian (*n* = 141)	
Average household income per month,^b^ €	6,340	4,752	307	22		
Poverty score^c^				40,4	69	
Household members	3.53	3.67	4.79	5.18	2.59	
Average education: father	University	University	Secondary	Primary	Secondary	
Average education: mother	University	University	Secondary	Primary	Secondary	
Country economy^d^	High-income	High-income	Lower-middle-income	Low-income	Lower-middle-income	
Country religion^e^	Church of Sweden (Lutheran; 54%), unspecified (37%)	Church of Sweden (Lutheran; 54%), unspecified^f^ (37%)	Buddhist (75%), Hindu (22%)	Christian (84%), Muslim (14%)	Christian (85%), none (8%)	

Note: All values are site averages with standard deviations in parentheses (unless otherwise stated). Some estimates were not derived from the data collected in the study (see a, e, and f). EPDS = Edinburgh Postnatal Depression Scale (Cox et al., 1987); WAEC = War and Adversity Exposure Checklist (Ibrahim et al., 2018); LS = life satisfaction (measured on a scale from 1 to 10 with the following question: “Taking all things together, how happy would you say you have been in the past 4 weeks?”); DRC = Democratic Republic of the Congo. ^a^Based on ethnicity in the current sample and religion per ethnicity reported in Gredebäck et al. (2023), in which 99% of Ngalop and Sharchop identified as Buddhist and 75% of Lhotsampa as Hindu. ^b^Transformed from the local currency to euros according to the exchange rate on March 26, 2024. ^c^Simple poverty scorecard (Uganda 2016/2017; https://www.simplepovertyscorecard.com/UGA-2016-ENG.pdf) measuring the likelihood that a family is poor on a scale from 0 (*high probability of poverty*) to 100 (*low probability of poverty*). ^d^Data taken from the World Bank (https://data.worldbank.org). ^e^Data taken from the World Factbook (https://www.cia.gov/the-world-factbook). ^f^Many Swedes belong to the Church of Sweden but are not religious, accounting for the difference between the overall country statistics and data.

### Materials and design

All stimuli were created by recording local models using a digital camera and editing the video clips to get identical onsets of the gaze shifts. The stimuli from Sweden 2 and Bhutan have been described previously (Astor et al., 2022) and were conceptually the same as but not identical to (i.e., recoded at a different time and with a different camera setup, six trials per infant vs. the current 16 trials per infant) the stimuli used for Sweden 1, Uganda, and Zimbabwe. Because stimuli and methods have already been presented for Sweden 2 and Bhutan, we hereafter describe only the stimuli and procedure for the novel recordings (Sweden 1, Uganda, and Zimbabwe).

**Fig. 2.**
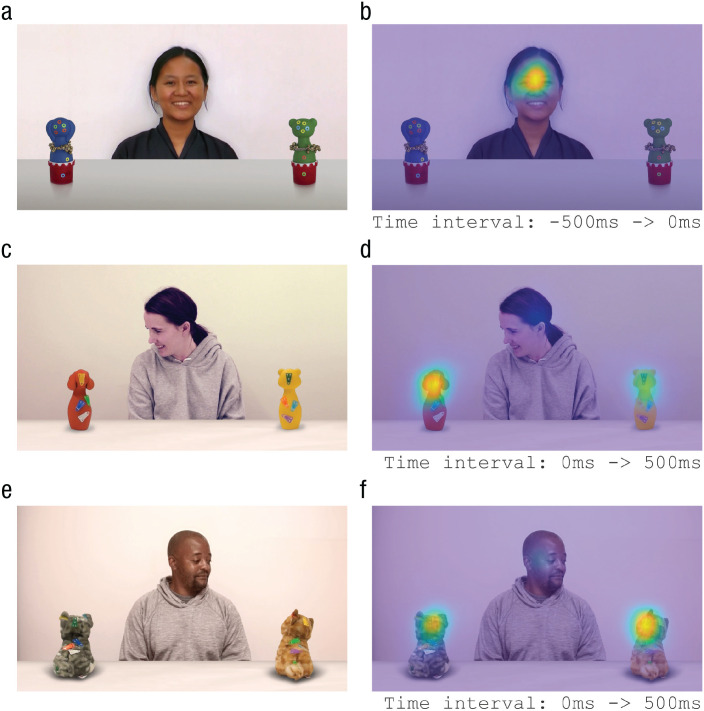
Stimuli and all novel gaze data. The stimuli included models from the local culture: (a) Bhutan (just before head turn and gaze shift), (c) Sweden (after head turn and gaze shift), and (e) Zimbabwe (after head turn and gaze shift). The corresponding images on the right show (b) a normalized heatmap of all infant gaze data in the time interval between −500 and 0 ms before infants’ gaze shifts and (d, f) similar heatmaps in the time interval between 0 and 500 ms after infants’ gaze shifts in the left and right conditions, respectively.

All infants performed an eye-tracking task in which they watched videos of an adult model. The model started with looking at the camera and greeting the child (except in the Sweden 2 sample, in which the greeting was replaced with a vocal-attention-cue “beep”) and thereafter moved their gaze to one of two objects located on a table in front of them. The infants typically watched the model and then moved their gaze to the object the model looked at (congruent gaze shift) or the other object (incongruent gaze shift). This was repeated over several trials and balanced important variables of the display, such as the direction of the model’s gaze shift (left/right), the gender of the model (male/female), and skin tone (same/different as majority population of the country). The stimuli always included culturally appropriate toys and models from the region in which the study was conducted while maintaining highly similar spatiotemporal properties such as the timing of events and the visual degrees covered by the actor and toys (Fig. 2; Movie S1 in the Supplemental Material available online). This experimental design is a gold standard for gaze-following research in infant eye-tracking studies (Del Bianco et al., 2019). To estimate the infants’ proneness to following other people’s gaze, a score was calculated either as a difference score (number of congruent minus incongruent gaze shifts) and reported as such in the main text and as a proportion score (number of congruent gaze shifts divided by the total number of gaze shifts), as reported in the Supplemental Material.

Each stimulus was an 8-s video clip with the model’s face at the top center of the video frame and one salient object at the bottom left and bottom right corners. The video clip started with the model looking into the camera and greeting the infant (0.0–3.0 s). The model then directed the head and gaze to one of the objects (3.0–4.0 s) and remained static until the end of the clip (4.0–8.0 s). In total, 16 videos were created: 2 female models × 2 skin tones × 2 gaze directions + 2 male models × 2 skin tones × 2 gaze directions (for a collage of all stimuli used in Sweden 1, Uganda, and Zimbabwe, see Movie S1). The movies were presented in a fixed order (female, light-skinned, congruent object to left; female, dark-skinned, congruent object to right; male, dark-skinned, congruent object to left; male, light-skinned, congruent object to left; female, light-skinned, congruent object to right; female, dark-skinned, congruent object to left; male, dark-skinned, congruent object to right; male, light-skinned, congruent object to right). The same model was never presented twice in a row, and the same sex, skin color, or gaze direction were never presented more than two times in a row. Stimuli presentation and eye-tracking recording were done using Tobii ProLab software (https://www.tobii.com).

### Procedure

After parents were informed about the study and signed consent, infants were seated on their caregivers’ laps in front of a computer monitor with an eye tracker attached under the screen (60-Hz Tobii Pro Nano eye tracker together with a 14-in. screen in all data sets except Sweden 1, in which a 60-Hz Tobii TX300 eye tracker together with a 24-in. screen were used). The position of the mother was adjusted so that the infant’s eyes were approximately 60 cm away from the screen. The mothers were instructed to refrain from interacting with their infants throughout the experiment and were monitored for compliance. After a standard Tobii 5-point calibration for infants the researcher began the experiment. The total experiment took approximately 9.7 min (*SD* = 2.5), and the gaze-following stimuli were interleaved with other tasks unrelated to the current study. If the infant became fussy or started crying the experiment was stopped, and any data collected up until that point were included in the analysis. After the recording all data were exported to text files, which were was analyzed using MATLAB r2022B (The MathWorks, Natick, MA) and the TimeStudio framework for time-series analysis (Nyström et al., 2016).

### Preprocessing and coding

To align the temporal resolution, we first resampled all time-series data to 60 Hz. A linear interpolation filled one- or two-sample gaps in the data, and high-frequency noise was removed using an I-VT fixation filter using Tobii software default settings (three-sample median filter type; maximum gap: 75 ms; velocity window: 20 ms; velocity threshold: 30 degrees/s; maximum angle between fixations: 0.5 degrees). Two-dimensional (2D) areas of interest (AOIs) were created using Voronoi patterns with points at the model, the two objects (congruent/incongruent), the corners of the screen, and the bottom center of the screen, and trials were segmented from the onset of models’ gaze shifts (time = 0 ms) to 5,000 ms after gaze-shift onset. Four types of trials were extracted: infants looking at the face of the model and then following the gaze to the congruent object (Type 1; total number of trials: 2,751; average: 3.400 trials per infant), infants looking at the face of the model and then at the incongruent object (Type 2; total number of trials: 1,667; average: 2.061 trials per infant), infants looking only at the face of the model (Type 3; total number of trials: 5,371: average: 9.027 trials per infant), and infants never looking at the face of the model (Type 4; total number of trials: 505; average: 0.849 trials per infant). Type 1 and 2 trials were used to calculate a difference score for which scores ≥ 1 indicated gaze following (Type 3 and 4 trials measured processes that do not relate to gaze following or were not possible to interpret using eye tracking). All raw data and fixation points were visually inspected overlaid on the 2D AOI plot and as time series for the *x* coordinates (see Fig. S1 in the Supplemental Material) to validate AOI classification and data quality (no trials were excluded).

Questionnaire data were collected using interviews with trained test leaders fluent in the local languages at all sites and covered basic background data (e.g., age, sex, income, education, family structure) as well as validated instruments tapping into aspects of the mothers’ mental health: the EPDS (Cox et al., 1987), War and Adversity Exposure Checklist (WAEC; Ibrahim et al., 2018), and the question “Taking all things together, how happy would you say you have been in the past 4 weeks?” on a scale from 1 (not at all) to 10 (very happy). The EPDS is a well-validated questionnaire that is in widespread use and that has been validated in nonpostnatal women (Cox et al., 1996). The scale consists of 10 items scored from 0 to 3, and we calculated the average of all items as our main variable of maternal well-being. The overall internal consistency in our samples was high, with an overall Cronbach’s α of .90 and a 95% confidence interval between .89 and .91 (Sweden .83, Bhutan .64, Uganda .74, Zimbabwe .83). The WAEC consists of 26 “yes/no questions” that assess mothers’ potentially traumatic events (without a specified time frame such as “in the last week/month/year”) and included questions such as “Have you ever witnessed someone being killed?” or “Have you ever been tortured?” We added 14 additional questions about climate and environmental shock exposure, such as “During the past 12 months, have you experienced drought?” or “During the past 12 months, have you experienced crop pests and disease?” These questions were selected by consensus among the authors for the purpose of stratifying groups in the gaze-following robustness assessment.

### Statistical analysis

All frequentist statistical analyses were performed in MATLAB r2022b using the ttest, ttest2, fitlm, and anova functions from the Statistical and Machine Learning Toolbox. We also performed a corresponding Bayesian test using the BayesFactor MATLAB package (https://zenodo.org/badge/latestdoi/162604707) to assess the strength of the null hypothesis. All Bayesian analyses used Cauchy-distributed Jeffreys-Zellner-Siow priors (*r* = .7071) on standardized effect sizes to avoid strongly biased results in favor of specific parameter values. Power analyses were performed after data collection using the MATLAB function sampsizepwr with default parameters (i.e., power for two-tailed *t* tests with α = .05). For the analysis of extreme aversive life events (Fig. 4), only participants at the Uganda site answered the WAEC questionnaire and could answer “yes.” All other participants were assumed to answer “no.”

## Results

**Fig. 3.**
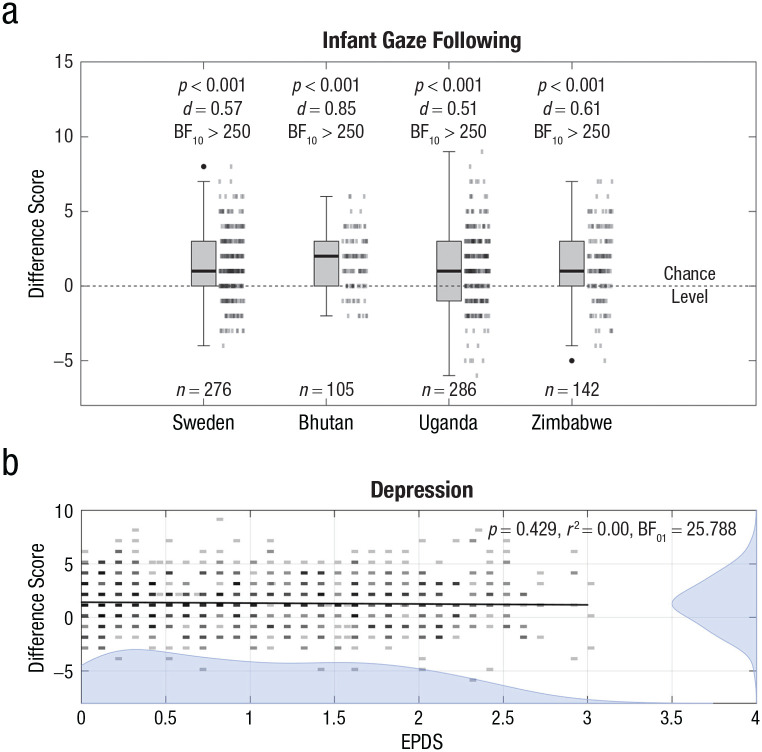
Gaze following at different locations. Gaze-following difference scores at (a) a group level were tested against the chance level (DS = 0) using two-tailed Student’s *t* tests with a Bonferroni correction (α) of .05, Cohen’s *d*, and Bayes factors. The boxplot displays the 25th to 75th percentiles and the median (black line). Whiskers denote the minimum and maximum values excluding outliers (black dots), and gray squares mark individual data points. The (b) scatterplot and regression line show the relationship between mothers’ self-rated depression score and gaze-following difference scores along with distribution estimates (blue area). EPDS = Edinburgh Postnatal Depression Scale.

A descriptive illustration of the general gaze-following patterns is shown in Figure 2, in which a clear gaze-following behavior can be seen across all participants, with significantly more looking time at the congruent object in all countries (Fig. 3a; Bonferroni Student’s *t* tests: all *p*s < .001, *d* = 0.51–0.85, BF_10_ > 250).

**Fig. 4.**
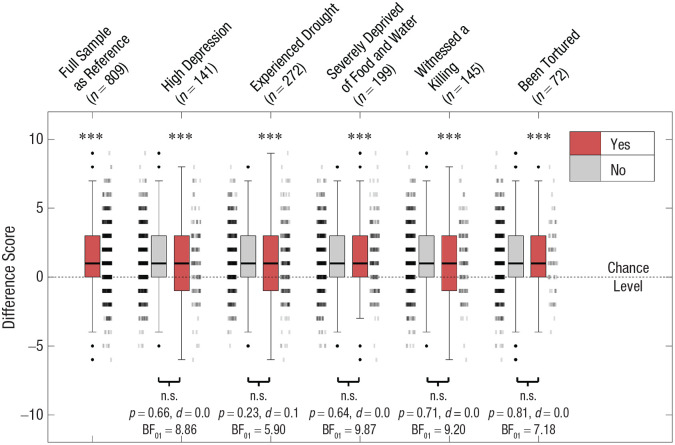
Effects of adverse and extreme life events. No significant differences were found between those who had a high score on the Edinburgh Postnatal Depression Scale (EPDS) or had experienced extreme life events, with low Cohen’s *d*s throughout and Bayes factors indicating support for the null hypothesis (statistics shown below the boxes). High depression was defined as an EPDS score > 2, corresponding to an EPDS raw score > 20. All groups, both “yes” and “no,” followed gaze significantly above chance (see the Supplemental Material). ***p < .001.

An *F* test including country of origin and child age showed no main or interaction effects—main effect of country origin: *F*(3, 801) = 0.654, *p* = .580; main effect of child age: *F*(1, 801) = 0.175, *p* = .676; interaction effect: *F*(3, 801) = 0.656, *p* = .580—suggesting that gaze following is similar across cultures, countries, and continents. In addition, a follow-up Bayesian analysis (assessing the probability/confidence of an outcome) showed very strong support for the null hypothesis for the full model compared with all alternate models (intercept only, country, child age, and country/child age: all BF_01_s > 250). All of these analyses were replicated with proportion scores (first gaze shift, looking time at congruent object, looking time at model) and number of gaze shifts between the model and the congruent object, with similar results suggesting that the findings were robust to variations in dependent measures (although one of 17 Bayesian analyses was inconclusive; see Fig. S3 in the Supplemental Material). As a final check, we investigated the risk of missing a true statistical effect (medium effect size, Cohen’s *d* = 0.5, based on default assumptions and real-world considerations, i.e., to find meaningful differences) by performing power analyses on the null results for two-tailed *t* tests with α = .05 and found statistical power to be > .99 (Fig. 3a) and between .85 and > .99 (Fig. 4), showing that we were very likely to detect statistical effects with the current sample sizes. Taken together, all main analyses unequivocally show robust effects of gaze following.

To assess whether gaze following was an experience-dependent or experience-expectant process, we analyzed gaze following in relation to the social family environment. We used the EPDS (Cox et al., 1987) as a proxy for the quality of the mother-infant interaction (Bjørnebekk et al., 2015; Goodman et al., 2020; Slomian et al., 2019; Zou et al., 2019) and found gaze following to be resilient to variations is maternal depression symptoms (*r*^2^ = .001; BF_01_ = 25.788; Fig. 3b). Similar results are reported with other mental-health indicators in Table S1 in the Supplemental Material, indicating that the effects were robust to variations in the questionnaires used. We then tested the effect of EPDS scores for each data set individually to determine whether any effect was masked by variability between countries. These models were not corrected for multiple testing to give conservative estimates of null results, and only the previously published result for Sweden 2 showed a significant EPDS effect, *F*(1, 101) = 4.084, *p* = .046. A follow-up Bayesian analysis of Sweden 2 gave a weak BF_01_ of 1.736 in favor of the null hypothesis (i.e., the BF_01_ supported the null hypothesis as 1.736 times more likely than the alternative hypothesis, but we considered these results inconclusive).

We also assessed adverse events in the mothers’ lives potentially affecting the quality of parent-child interaction by stratifying groups on the most extreme items in the WAEC (Ibrahim et al., 2018). Infants followed gaze in all groups, irrespective of whether the mother had experienced war-related potentially traumatic events such as witnessing a killing (*n* = 145, *M* = 1.276, *SD* = 2.476) or having been tortured (*n* = 72, *M* = 1.403, *SD* = 2.430), events related to global warming such as drought (*n* = 272, *M* = 1.202, *SD* = 2.470), or severe deprivation of food and water (*n* = 199, *M* = 1.407, *SD* = 2.395), values that were very similar to the average gaze following across all sites (*n* = 809, *M* = 1.340, *SD* = 2.312). We did not find any significant differences between groups regardless of whether we stratified the groups on the basis of high EPDS (> 2, equivalent to a raw score > 20), climate adversities, starvation, or war-related events (Fig. 4). Follow-up analyses using Bayesian statistics gave BF_01_s ranging from 5.9 to 9.9, which shows the strength of the null hypothesis and that the groups were indeed very similar.

To test the robustness of early gaze following and further test the effect of the EPDS, we stratified two groups on the basis of high EPDS scores (≥ 2; Fig. 4). This analysis was not significant when tested with an independent *t* test, *t*(807) = 0.438, *p* = .662, *d* = 0.04, and a BF_01_ of 8.859 (“yes”: *n* = 141; “no”: *n* = 668) showed moderate support for the null hypothesis.

Similar analyses (with similar results) were performed for extreme aversive events: “Experienced drought” (“yes” *n* = 272, “no” *n* = 537), *t*(807) = 1.206, *p* = .228, *d* = 0.09, BF_01_ = 5.904; “Severely deprived of food and water” (“yes” *n* = 199, “no” *n* = 610), *t*(807) = −0.471, *p* = .638, *d* = 0.04, BF_01_ = 9.867; “Witnessed a killing” (“yes” *n* = 145, “no” *n* = 664), *t*(807) = 0.368, *p* = .713, *d* = 0.03, BF_01_ = 9.201; and “Been tortured” (“yes” *n* = 72, “no” *n* = 737), *t*(807) = −0.242, *p* = .809, *d* = 0.03, BF_01_ = 7.183.

It is worth noting that all stratified groups had gaze-following difference scores well above chance when tested against zero despite having smaller sample sizes—“High EPDS”: “yes,” *t*(140) = 5.763, *p* < .001, “no” *t*(667) = 15.592, *p* < .001; “Experienced drought”: “yes” *t*(271) = 8.026, *p* < .001, “no” *t*(536) = 14.670, *p* < .001; “Severely deprived of food and water”: “yes” *t*(198) = 8.286, *p* < .001, “no” *t*(609) = 14.241, *p* < .001; “Witnessed a killing”: “yes” *t*(144) = 6.204, *p* < .001, “no” *t*(663) = 15.326, *p* < .001; “Been tortured”: “yes” *t*(71) = 4.898, *p* < .001, “no” *t*(736) = 15.730, *p* < .001. All *p* values were Bonferroni corrected for 10 tests (five “yes” and five “no”).

Many of the refugees in Uganda have faced several aversive events, and it is possible that the same participants are always grouped together. To assess the overlap between participants in the different extreme groups, we calculated the percentage overlap between two groups by dividing the number of “yes” participants in both groups with the number of “yes” participants in any of the two groups for all combinations of extreme groups (see Table S2 in the Supplemental Material). The percentage range was between 24% and 72%, indicating that the extreme groups represent different subsets of participants.

## Discussion

Nine-month-old infants follow gaze to a similar extent regardless of their upbringing in locations as diverse as Bhutan, Sweden, Uganda, or Zimbabwe. This ability does not appear to depend on parental functioning because it is robust to variations in maternal mental health and horrific experiences related to war and global warming. This suggests that gaze following is a universal, experience-expectant process that is resilient to adversity and cultural variations in rearing practices (as previously suggested by Hernik & Broesch, 2019; Spelke, 2022). In interpreting these findings, it is important to note that our claims of universality extend only to distal social referencing. There are likely other culture-specific cues that express jointness and attention to external events (such as closeness) that exist in addition to the distal referential action investigated here (Bard et al., 2021).

We have previously argued for the alternative hypothesis—that gaze following depends on high-quality social interactions and good maternal mental health (Astor et al., 2020, 2022). The data used to draw this inference are included in the current data (Sweden 2). As can be seen in Figure S2 in the Supplemental Material, there is a weak but significant correlation in this sample, connecting maternal mental health and infant gaze following. When critically evaluating this claim by zooming out to a global perspective, and adding a second Swedish sample, an alternative picture emerges—the opposite of what we, and others, have proposed in the past. This highlights the need for both large sample sizes and a global approach to child development that assess robustness across samples, contexts, and with individual variations that capture the breadth of human experiences.

Before concluding, a series of caveats should be mentioned. We inferred properties of parent-child interactions from the mental health of mothers. This inference did not hold for all individuals, but the associations were sufficiently robust on a group level, and over large samples, to motivate the taken approach (Bjørnebekk et al., 2015; Goodman et al., 2020; Slomian et al., 2019; Zou et al., 2019). Recording and coding parent-child interactions would have added unique value (Keller, 2007) but would likely not have changed our conclusions and was simply not feasible given the scale of the study.

It is known that children with symptoms of autism-spectrum disorder (ASD) are less likely to follow others’ gaze (Del Bianco et al., 2019), and this was something that we did not control for. In this regard it is important to note that infant studies have reported that even infants who were later diagnosed with ASD followed gaze at the age we assessed here (Nyström et al., 2019), further cementing the notion that gaze following, in infancy, is an experience-expectant universal process available to the vast majority of infants.

We demonstrated that gaze following was highly consistent across contexts. The two most extreme contexts are perhaps high-income Swedish families with a high degree of well-being and low-income refugees in Uganda with poor mental health and often severe war-related experiences. Both on a group and individual level these diverse experiences did not appear to alter infants’ tendency to follow gaze. We suggest that the current results support claims of universality. It is always difficult to demonstrate that there are no exceptions to a rule, but given the diversity of settings and the breadth of human experiences sampled we argue that universality is the most parsimonious explanation of currently available data.

Along the same lines, we note that the data include a lot of variability. In fact, infants followed gaze on an average of 3.4 of 16 trials; the remaining trials included those with a gaze shift to the incongruent object but also trials in which infants did not attend at all or fixate the face without making a gaze shift to either object. As such gaze following was not an automatic reflex-like process triggered by environmental cues. We attribute this variability to random noise and lack of attention, naturally occurring in all infant research. But it is important to acknowledge that this variation could be generated by unknown experiential moderators not currently identified.

With these caveats in mind, the current results were unexpected yet very clear. They suggest that infant gaze following is an experience-expectant, or core-knowledge (Spelke, 2022), process that emerges in a similar manner in most infants. This skill helps infants engage in triadic social interactions and develop more advanced forms of social cognition such as theory of mind, a more complex process that we know is based on individual experiences, cultural contexts, and an active interpretation of the social context in which children grow up (Wellman, 2018). However, we find it hopeful that the foundational processes that make this possible are universal and present even under very challenging conditions.

## Supplemental Material

sj-docx-1-pss-10.1177_09567976251331042 – Supplemental material for Infant Gaze Following Is Stable Across Markedly Different Cultures and Resilient to Family Adversities Associated With War and Climate Change Supplemental material, sj-docx-1-pss-10.1177_09567976251331042 for Infant Gaze Following Is Stable Across Markedly Different Cultures and Resilient to Family Adversities Associated With War and Climate Change by Gustaf Gredebäck, Kim Astor, Herbert Ainamani, Linda van den Berg, Linda Forssman, Jonathan Hall, Joshua Juvrud, Ben Kenward, Samson Mhizha, Samson Wangchuk and Pär Nyström in Psychological Science

sj-png-2-pss-10.1177_09567976251331042 – Supplemental material for Infant Gaze Following Is Stable Across Markedly Different Cultures and Resilient to Family Adversities Associated With War and Climate Change Supplemental material, sj-png-2-pss-10.1177_09567976251331042 for Infant Gaze Following Is Stable Across Markedly Different Cultures and Resilient to Family Adversities Associated With War and Climate Change by Gustaf Gredebäck, Kim Astor, Herbert Ainamani, Linda van den Berg, Linda Forssman, Jonathan Hall, Joshua Juvrud, Ben Kenward, Samson Mhizha, Samson Wangchuk and Pär Nyström in Psychological Science

sj-png-3-pss-10.1177_09567976251331042 – Supplemental material for Infant Gaze Following Is Stable Across Markedly Different Cultures and Resilient to Family Adversities Associated With War and Climate Change Supplemental material, sj-png-3-pss-10.1177_09567976251331042 for Infant Gaze Following Is Stable Across Markedly Different Cultures and Resilient to Family Adversities Associated With War and Climate Change by Gustaf Gredebäck, Kim Astor, Herbert Ainamani, Linda van den Berg, Linda Forssman, Jonathan Hall, Joshua Juvrud, Ben Kenward, Samson Mhizha, Samson Wangchuk and Pär Nyström in Psychological Science

sj-png-4-pss-10.1177_09567976251331042 – Supplemental material for Infant Gaze Following Is Stable Across Markedly Different Cultures and Resilient to Family Adversities Associated With War and Climate Change Supplemental material, sj-png-4-pss-10.1177_09567976251331042 for Infant Gaze Following Is Stable Across Markedly Different Cultures and Resilient to Family Adversities Associated With War and Climate Change by Gustaf Gredebäck, Kim Astor, Herbert Ainamani, Linda van den Berg, Linda Forssman, Jonathan Hall, Joshua Juvrud, Ben Kenward, Samson Mhizha, Samson Wangchuk and Pär Nyström in Psychological Science

sj-png-5-pss-10.1177_09567976251331042 – Supplemental material for Infant Gaze Following Is Stable Across Markedly Different Cultures and Resilient to Family Adversities Associated With War and Climate Change Supplemental material, sj-png-5-pss-10.1177_09567976251331042 for Infant Gaze Following Is Stable Across Markedly Different Cultures and Resilient to Family Adversities Associated With War and Climate Change by Gustaf Gredebäck, Kim Astor, Herbert Ainamani, Linda van den Berg, Linda Forssman, Jonathan Hall, Joshua Juvrud, Ben Kenward, Samson Mhizha, Samson Wangchuk and Pär Nyström in Psychological Science

sj-png-6-pss-10.1177_09567976251331042 – Supplemental material for Infant Gaze Following Is Stable Across Markedly Different Cultures and Resilient to Family Adversities Associated With War and Climate Change Supplemental material, sj-png-6-pss-10.1177_09567976251331042 for Infant Gaze Following Is Stable Across Markedly Different Cultures and Resilient to Family Adversities Associated With War and Climate Change by Gustaf Gredebäck, Kim Astor, Herbert Ainamani, Linda van den Berg, Linda Forssman, Jonathan Hall, Joshua Juvrud, Ben Kenward, Samson Mhizha, Samson Wangchuk and Pär Nyström in Psychological Science

sj-png-7-pss-10.1177_09567976251331042 – Supplemental material for Infant Gaze Following Is Stable Across Markedly Different Cultures and Resilient to Family Adversities Associated With War and Climate Change Supplemental material, sj-png-7-pss-10.1177_09567976251331042 for Infant Gaze Following Is Stable Across Markedly Different Cultures and Resilient to Family Adversities Associated With War and Climate Change by Gustaf Gredebäck, Kim Astor, Herbert Ainamani, Linda van den Berg, Linda Forssman, Jonathan Hall, Joshua Juvrud, Ben Kenward, Samson Mhizha, Samson Wangchuk and Pär Nyström in Psychological Science

sj-png-8-pss-10.1177_09567976251331042 – Supplemental material for Infant Gaze Following Is Stable Across Markedly Different Cultures and Resilient to Family Adversities Associated With War and Climate Change Supplemental material, sj-png-8-pss-10.1177_09567976251331042 for Infant Gaze Following Is Stable Across Markedly Different Cultures and Resilient to Family Adversities Associated With War and Climate Change by Gustaf Gredebäck, Kim Astor, Herbert Ainamani, Linda van den Berg, Linda Forssman, Jonathan Hall, Joshua Juvrud, Ben Kenward, Samson Mhizha, Samson Wangchuk and Pär Nyström in Psychological Science

sj-png-9-pss-10.1177_09567976251331042 – Supplemental material for Infant Gaze Following Is Stable Across Markedly Different Cultures and Resilient to Family Adversities Associated With War and Climate Change Supplemental material, sj-png-9-pss-10.1177_09567976251331042 for Infant Gaze Following Is Stable Across Markedly Different Cultures and Resilient to Family Adversities Associated With War and Climate Change by Gustaf Gredebäck, Kim Astor, Herbert Ainamani, Linda van den Berg, Linda Forssman, Jonathan Hall, Joshua Juvrud, Ben Kenward, Samson Mhizha, Samson Wangchuk and Pär Nyström in Psychological Science

sj-png-10-pss-10.1177_09567976251331042 – Supplemental material for Infant Gaze Following Is Stable Across Markedly Different Cultures and Resilient to Family Adversities Associated With War and Climate Change Supplemental material, sj-png-10-pss-10.1177_09567976251331042 for Infant Gaze Following Is Stable Across Markedly Different Cultures and Resilient to Family Adversities Associated With War and Climate Change by Gustaf Gredebäck, Kim Astor, Herbert Ainamani, Linda van den Berg, Linda Forssman, Jonathan Hall, Joshua Juvrud, Ben Kenward, Samson Mhizha, Samson Wangchuk and Pär Nyström in Psychological Science

sj-png-11-pss-10.1177_09567976251331042 – Supplemental material for Infant Gaze Following Is Stable Across Markedly Different Cultures and Resilient to Family Adversities Associated With War and Climate Change Supplemental material, sj-png-11-pss-10.1177_09567976251331042 for Infant Gaze Following Is Stable Across Markedly Different Cultures and Resilient to Family Adversities Associated With War and Climate Change by Gustaf Gredebäck, Kim Astor, Herbert Ainamani, Linda van den Berg, Linda Forssman, Jonathan Hall, Joshua Juvrud, Ben Kenward, Samson Mhizha, Samson Wangchuk and Pär Nyström in Psychological Science

sj-png-12-pss-10.1177_09567976251331042 – Supplemental material for Infant Gaze Following Is Stable Across Markedly Different Cultures and Resilient to Family Adversities Associated With War and Climate Change Supplemental material, sj-png-12-pss-10.1177_09567976251331042 for Infant Gaze Following Is Stable Across Markedly Different Cultures and Resilient to Family Adversities Associated With War and Climate Change by Gustaf Gredebäck, Kim Astor, Herbert Ainamani, Linda van den Berg, Linda Forssman, Jonathan Hall, Joshua Juvrud, Ben Kenward, Samson Mhizha, Samson Wangchuk and Pär Nyström in Psychological Science

## Transparency

*Action Editor:* Louis J. Moses

*Editor:* Simine Vazire

*Author Contributions*

**Gustaf Gredebäck:** Conceptualization; Funding acquisition; Methodology; Project administration; Supervision; Writing – original draft; Writing – review & editing.

**Kim Astor:** Data curation; Investigation; Project administration; Visualization; Writing – review & editing.

**Herbert Ainamani:** Conceptualization; Project administration; Supervision; Writing – review & editing.

**Linda van den Berg:** Data curation; Investigation; Project administration; Writing – review & editing.

**Linda Forssman:** Data curation; Project administration; Writing – review & editing.

**Jonathan Hall:** Conceptualization; Project administration; Visualization; Writing – review & editing.

**Joshua Juvrud:** Data curation; Investigation; Project administration; Writing – review & editing.

**Ben Kenward:** Methodology; Writing – review & editing.

**Samson Mhizha:** Conceptualization; Project administration; Writing – review & editing.

**Wangchuk:** Conceptualization; Project administration; Writing – review & editing.

**Pär Nyström:** Conceptualization; Data curation; Formal analysis; Project administration; Software; Validation; Visualization; Writing – original draft; Writing – review & editing.

**Artificial Intelligence:** No AI-assisted technologies were used in this research or the creation of this article.

**Ethics:** This research received approval from a local ethics board at all locations.

**Open Practices:** Study disclosures. Preregistration: No aspect of the study was preregistered. The project is described at https://osf.io/t7rxn/files/osfstorage. Materials: All study materials are publicly available (https://osf.io/j69bm/files/osfstorage). Data: All primary gaze data and questionnaire primary data used in this publication are publicly available (https://osf.io/dx3zv/files/osfstorage). Analysis scripts: All analysis scripts are publicly available (https://osf.io/dx3zv/files/osfstorage). Computational reproducibility: Because of resource constraints, the journal’s STAR team did not complete a reproducibility check for this manuscript.
