# Supplementary material for: Infant Gaze Following Is Stable Across Markedly Different Cultures and Resilient to Family Adversities Associated With War and Climate Change
Source: Psychol Sci. 2025 Apr 21;36(4):296–307. doi: 10.1177/09567976251331042 (PMC13428878; doi:10.1177/09567976251331042)
Supplement: sj-docx-1-pss-10.1177_09567976251331042 – Supplemental material for Infant Gaze Following Is Stable Across Markedly Different Cultures and Resilient to Family Adversities Associated With War and Climate Change [file sj-docx-1-pss-10.1177_09567976251331042.docx]

Supplementary Material for

Gaze following in infancy, a robust experience-expectant universal social ability.

**Authors:**

Gustaf Gredebäck^1^*, Kim Astor^1^, Linda van den Berg^1^, Joshua Juvrud^2^, Linda Forssman^1^, Ben Kenward^1^, Herbert Ainamani^3^, Jonathan Hall^4^, Samson Mhizha^5^, Wangchuk^6^, Pär Nyström^1^

*Corresponding author. [gustaf.gredeback@psyk.uu.se](mailto:gustaf.gredeback@psyk.uu.se)

This PDF file includes:

Materials and Methods

Supplementary text

Figs. S1 to S5

References

Supplementary methods

We used the EPDS questionnaire as our proxy for quality of mother-child interaction because this questionnaire was available in all datasets. To eliminate the possibility that our results are specific to this questionnaire and to show the relation between the EPDS and our other mental health questionnaires, we calculated a correlation matrix (Table S1). The amount of overlap between different potentially traumatic events is depicted in Table S2. Scatterplots illustrating the relation between gaze following and EPDS score, separate for each site, can be observed in Figure S2.

**Exploratory analyses.** In addition to the analyses above, we performed a number of exploratory analyses not crucial to our main conclusions. Most of these were identical to the analyses in the main manuscript, but with the dependent variable being a proportion score instead of a difference score: nrCongruentTrials/(nrCongruentTrials+nrIncongruentTrials). One advantage of using proportions is that numbers of valid trials are normalized; i.e. an infant that always follow gaze will get a score of 1 regardless of number of trials presented or watched. However, the fidelity of the proportion score is dependent on the number of trials, which often results in non-normal distributions. In our figures this can be spotted as many minimum and maximum values (either 0 or 1 proportions), and a separate cluster of values in-between. Researchers tend to favor either difference scores or proportion scores, but our results with proportion scores (Fig. S3a and S4a) show that the results are very similar to the main results (Fig. 3 and 4).

To further investigate the looking behaviors during the stimuli we performed similar analyses for other measures: proportions looking time at the congruent object, proportions looking time at the model, and the number of gaze shifts between the model and the congruent object (Fig. S3b-d and S4b-d; although 4 of 32 Bayesian analyses were inconclusive). The results from these analyses show no results conflicting with the main analyses, further enhancing the robustness of the main findings.

To explore the robustness over trials we calculated new difference scores including an increasing number of presented stimuli (Fig. S5A, green). Trial level data were only available for the novel datasets (Sweden1, Uganda and Zimbabwe), and included 16 trials for each infant. The group’s difference scores were compared against 0 using Student’s t-tests with Bonferroni correction for 16 tests, and all tests showed significant gaze following at *p*<0.001. I.e. there is robust gaze following already at the first presented trial at the group level, which increase with the number of presented trials. Next, we tested whether there were habituation effects by reversing the order of trials included in the analysis (Fig. S5A, red line). The results showed that the difference score did not differ from 0 (with a *p*=0.117, uncorrected *p*=0.007) when only the last trial was included. When including two or more trials the p-values was always below 0.001. Overall, these results suggest that infants’ gaze following is not habituated over repeated stimuli presentations, which aligns with the universality and robustness results of our other analyses.

While the group level data is unambiguous, the individuals’ data contains much variability (Fig. S5B). Although it is difficult to interpret a negative difference score, a common conception is that it reflects the stochastic nature of infants’ scan paths. With all trials included (i.e. at trial 16), 377 infants had a positive difference score, 85 had a score of 0, and 136 infants had a negative score. Because of this variation we caution against using small sample sizes, and advise against interpreting individual scores. However, at a group level the effect of gaze following is remarkably robust.

**Supplementary Movie. Stimuli used in Sweden, Uganda and Zimbabwe combined into one movie display.**

This movie can also be found at <https://osf.io/j9rhf/> wi

***Fig S1.*** *Example of the visual inspection procedure for all trials. Voronoi AOIs covered the model’s face (blue area), the congruent object (green area), and the incongruent area (red area). The time series in the bottom part of each panel shows the gaze X-coordinate, with raw data as a thin grey line and fixation filtered X-coordinate as a thick line colored according to the visited AOI. Top left panel shows a congruent trial, top right panel shows an incongruent trial, and bottom center panel shows an invalid trial where the infant doesn’t follow gaze and only looks at the model.*

***Fig S2.*** *Scatter plots of gaze following difference scores across EPDS depression scores. Black line is regression line and blue areas are distribution estimates (computed probability density estimates from the Matlab function ksdensity with default settings). Regression p-values, r^2^ effect sizes, and Bayes factors showing strength of the null hypothesis are shown in top right corner of each plot.*

***Fig S3a.*** *Same figure as in Fig. 3 in main manuscript but with proportion scores instead of difference scores: nrCongruentTrials/(nrCongruentTrials + nrIncongruentTrials).*

***Fig S3b.*** *Same figure as in Fig. 3 in main manuscript but with proportion looking time at the target instead of difference scores: lookingtimeCongruent/(lookingtimeCongruent+lookingtimeIncongruent). Note that Bhutan and Sweden2 are not included due to lack of raw eye tracking data.*

***Fig S3c.*** *Same figure as in Fig. 3 in main manuscript but with proportion looking time at the model instead of difference scores: lookingtimeModel/(lookingtimeCongruent+lookingtimeIncongruent). Note that Bhutan and Sweden2 are not included due to lack of raw eye tracking data.*

***Fig S4a.*** *Same figure as in Fig. 4 in main manuscript but with proportion scores instead of difference scores: nrCongruentTrials/(nrCongruentTrials+nrIncongruentTrials). Asterixes above the boxes refer to overall gaze following in the Yes groups (all groups statistically follow gaze, p<0.001). The statistical comparison between the yes and no groups is presented below the boxes (p-values, Cohen’s d effect sizes, and Bayes factor for the null hypothesis; all groups are non-significant with Bayes factors supporting the null hypothesis, except “Experienced drought”, which is significant with a less than small effect size, and a Bayes factor suggesting that this result is inconclusive).*

***Fig S4b.*** *Same figure as in Fig. 4 in main manuscript but with proportion looking times instead of difference scores: lookingtimeCongruent/(lookingtimeCongruent+lookingtimeIncongruent). Asterixes above the boxes refer to overall gaze following (all groups look more at the congruent object). The statistical comparison between the yes and no groups is presented below the boxes (p-values, Cohen’s d effect sizes, and Bayes factor for the null hypothesis).*

***Fig S4c.*** *Same figure as in Fig. 4 in main manuscript but with proportion looking at the model instead of difference scores: lookingtimeModel/(lookingtimeModel+lookingtimeCongruent+lookingtimeIncongruent). Asterixes above the boxes refer to overall gaze following (all groups statistically look mostly at the model). The statistical comparison between the yes and no groups is presented below the boxes (p-values, Cohen’s d effect sizes, and Bayes factor for the null hypothesis).*

***Fig S4d.*** *Same figure as in Fig. 4 in main manuscript but with number of gaze shifts between the model and the congruent object. The statistical comparison between the yes and no groups is presented below the boxes (p-values, Cohen’s d effect sizes, and Bayes factor for the null hypothesis; all groups are non-significant with Bayes factors supporting the null hypothesis, except “High depression” and “Experienced drought”, which are significant but have very small effect sizes, and Bayes factors suggesting that these results are inconclusive).*

***Fig S5. (A)*** *The difference score with different numbers of trials included (i.e. at x=1 the difference score is only calculated from the first presented stimuli, and at x=16 are all trials included). Left panel shows group data, and the red line shows the same analysis but with the trial order reversed (i.e. x=1 is the last presented trial, x=2 are the difference score of the last 2 presented trial etc). Shaded areas represent 95% confidence intervals.* ***(B)*** *Individual accumulating difference scores, with one transparent line for each infant. Lines are randomly offset for illustrational purposes.*

***Table S1. Correlation between gaze following and questionnaires.*** **p<0.05, **p<0.01, ***p<0.001*

|  | **Gaze following** | **Depression** | **Life satisfaction** |
| --- | --- | --- | --- |
| **Gaze following** | - | - | - |
| **Depression** | -0.028 n=793 | - | - |
| **Life satisfaction** | 0.006 n=578 | -0.706*** n=577 | - |

***Table S2. Percent overlapping participants between extreme groups.***

|  | **High EPDS** | **Drought** | **Food and water shortage** | **Witnessed a killing** | **Been tortured** |
| --- | --- | --- | --- | --- | --- |
| **High EPDS** | 100% |  |  |  |  |
| **Drought** | 46% | 100% |  |  |  |
| **Food and water shortage** | 42% | 69% | 100% |  |  |
| **Witnessed a killing** | 36% | 50% | 72% | 100% |  |
| **Been tortured** | 28% | 24% | 35% | 40% | 100% |
