# Supplementary figures and images for: Infant Gaze Following Is Stable Across Markedly Different Cultures and Resilient to Family Adversities Associated With War and Climate Change
Source: Psychol Sci. 2025 Apr 21;36(4):296–307. doi: 10.1177/09567976251331042 (PMC13428878; doi:10.1177/09567976251331042)

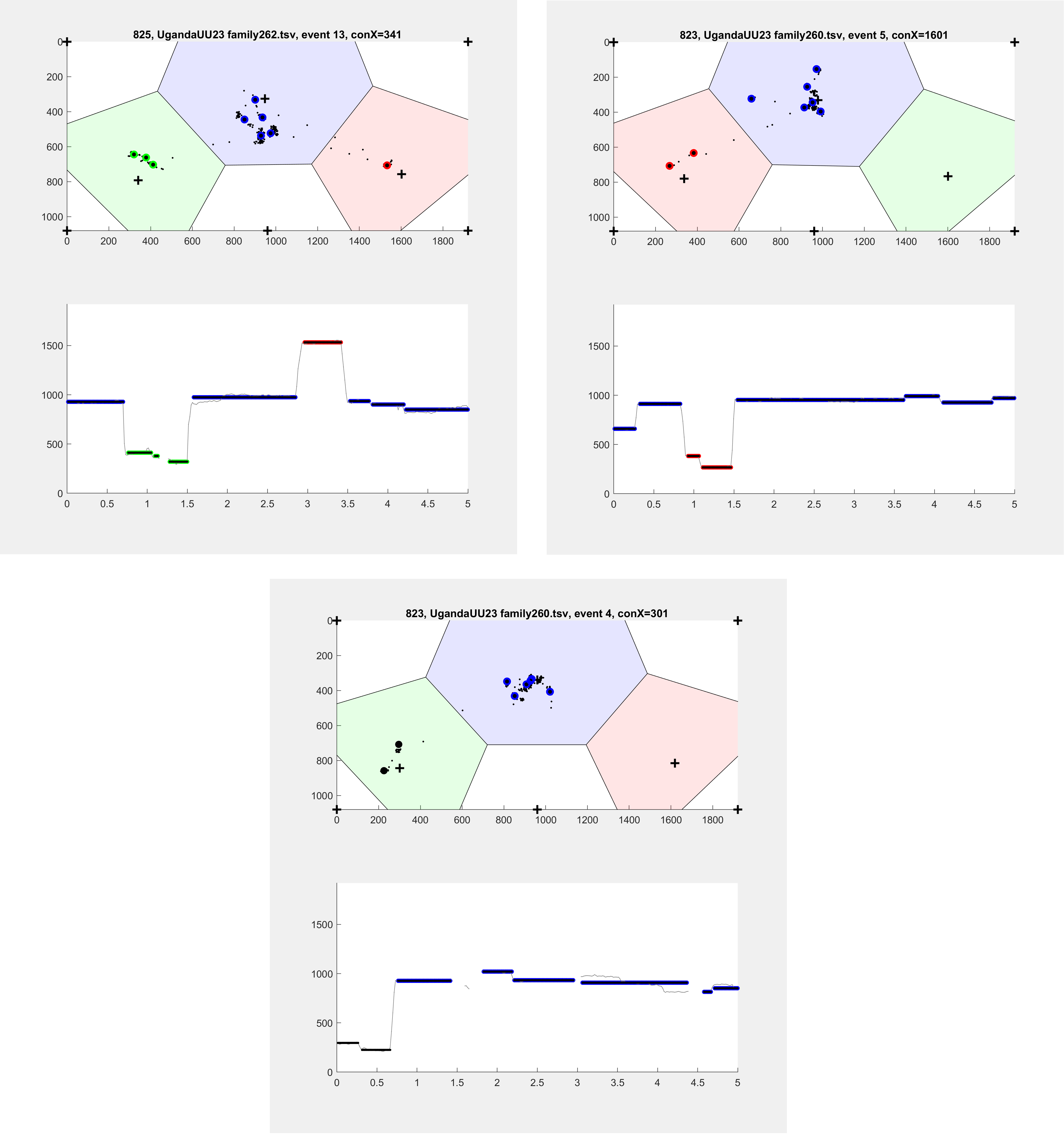

Supplement: sj-png-2-pss-10.1177_09567976251331042 – Supplemental material for Infant Gaze Following Is Stable Across Markedly Different Cultures and Resilient to Family Adversities Associated With War and Climate Change [file sj-png-2-pss-10.1177_09567976251331042.png]

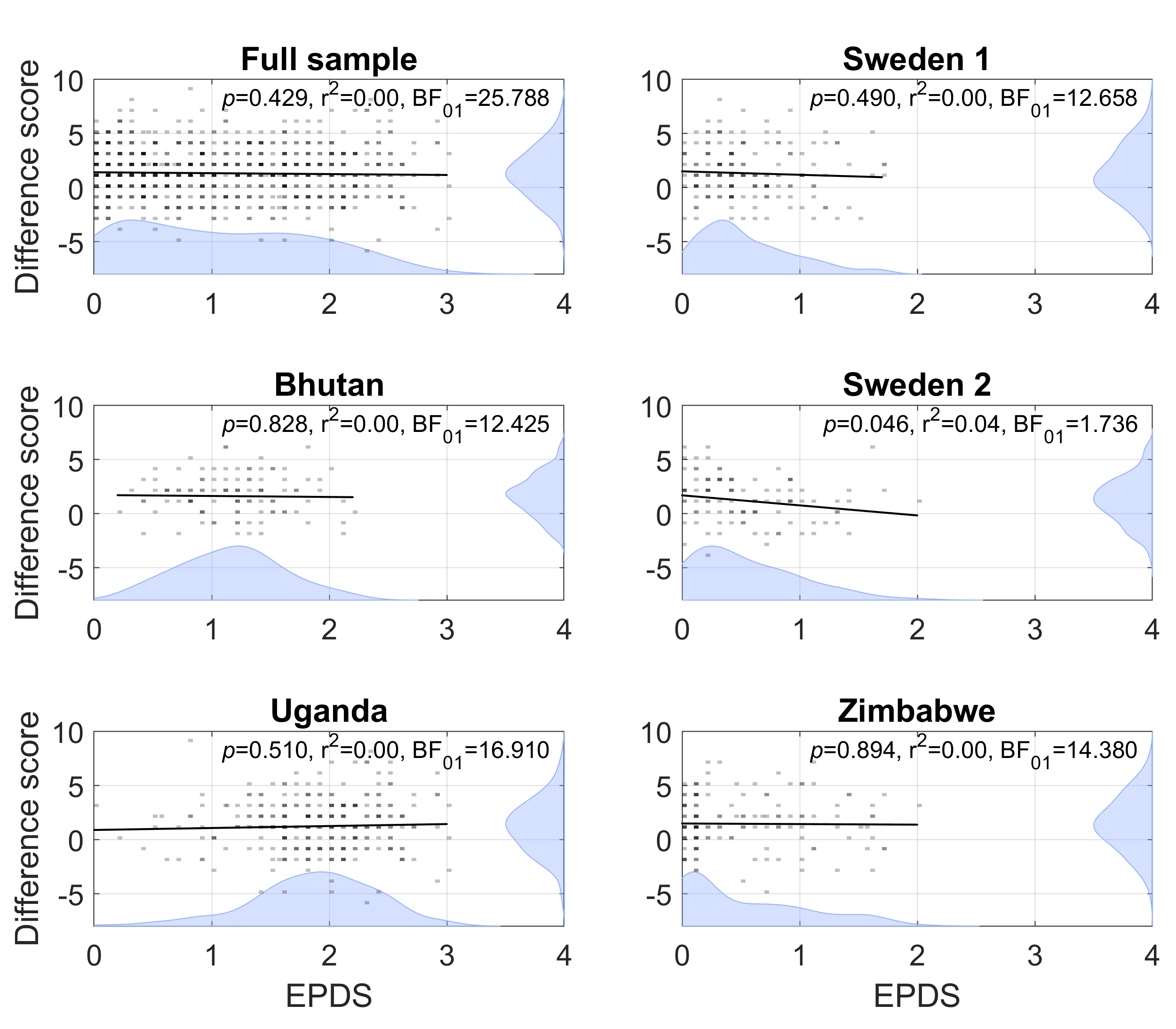

Supplement: sj-png-3-pss-10.1177_09567976251331042 – Supplemental material for Infant Gaze Following Is Stable Across Markedly Different Cultures and Resilient to Family Adversities Associated With War and Climate Change [file sj-png-3-pss-10.1177_09567976251331042.png]

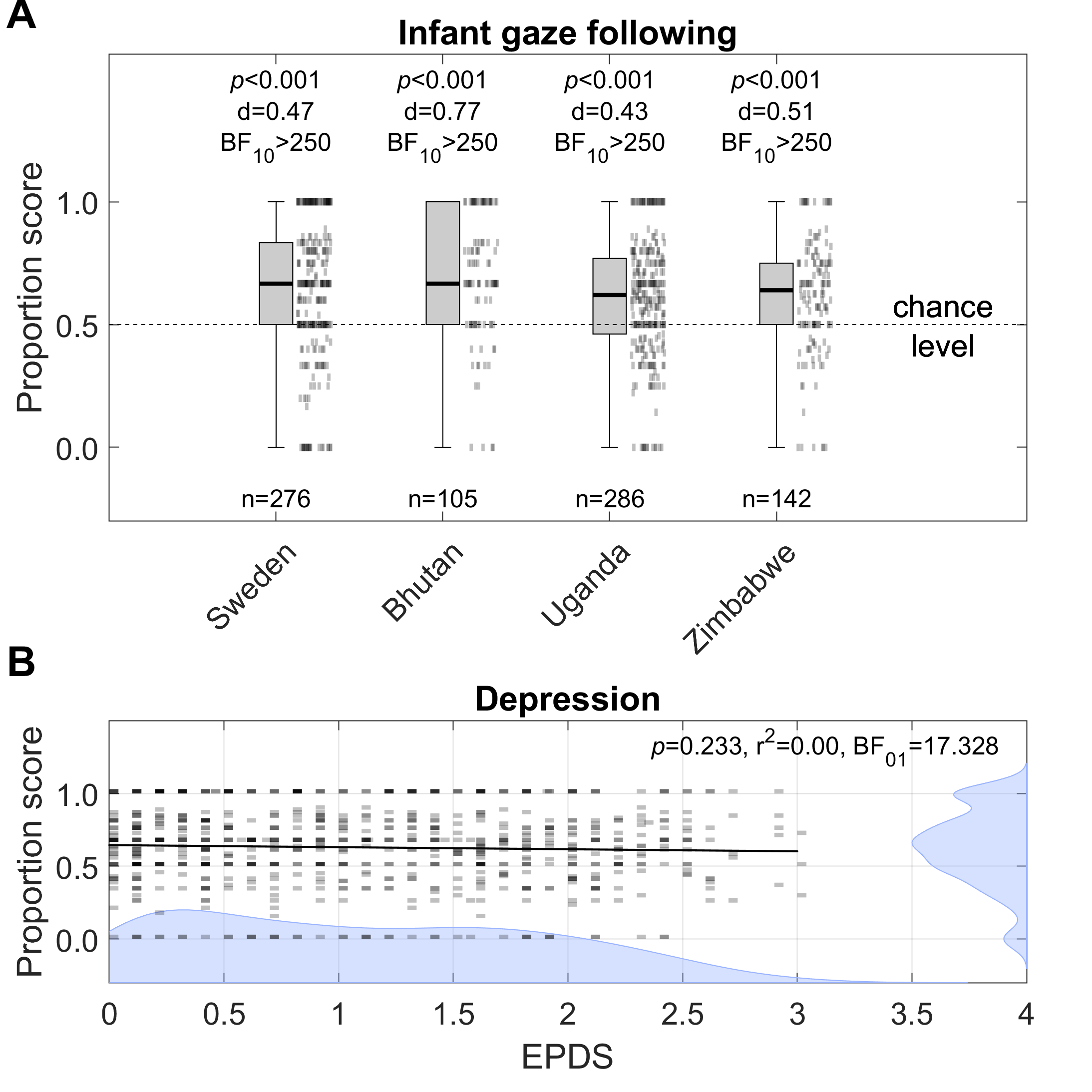

Supplement: sj-png-4-pss-10.1177_09567976251331042 – Supplemental material for Infant Gaze Following Is Stable Across Markedly Different Cultures and Resilient to Family Adversities Associated With War and Climate Change [file sj-png-4-pss-10.1177_09567976251331042.png]

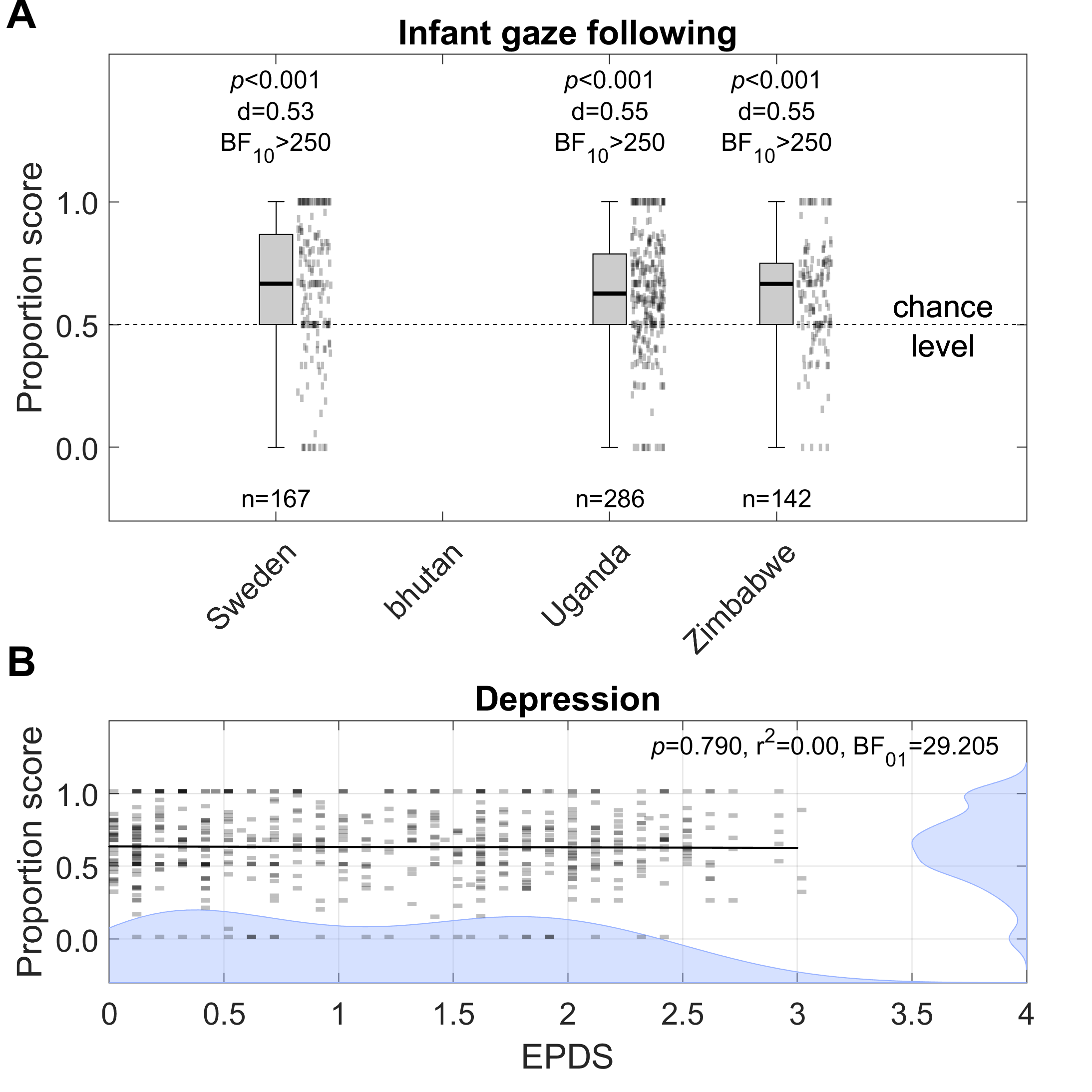

Supplement: sj-png-5-pss-10.1177_09567976251331042 – Supplemental material for Infant Gaze Following Is Stable Across Markedly Different Cultures and Resilient to Family Adversities Associated With War and Climate Change [file sj-png-5-pss-10.1177_09567976251331042.png]

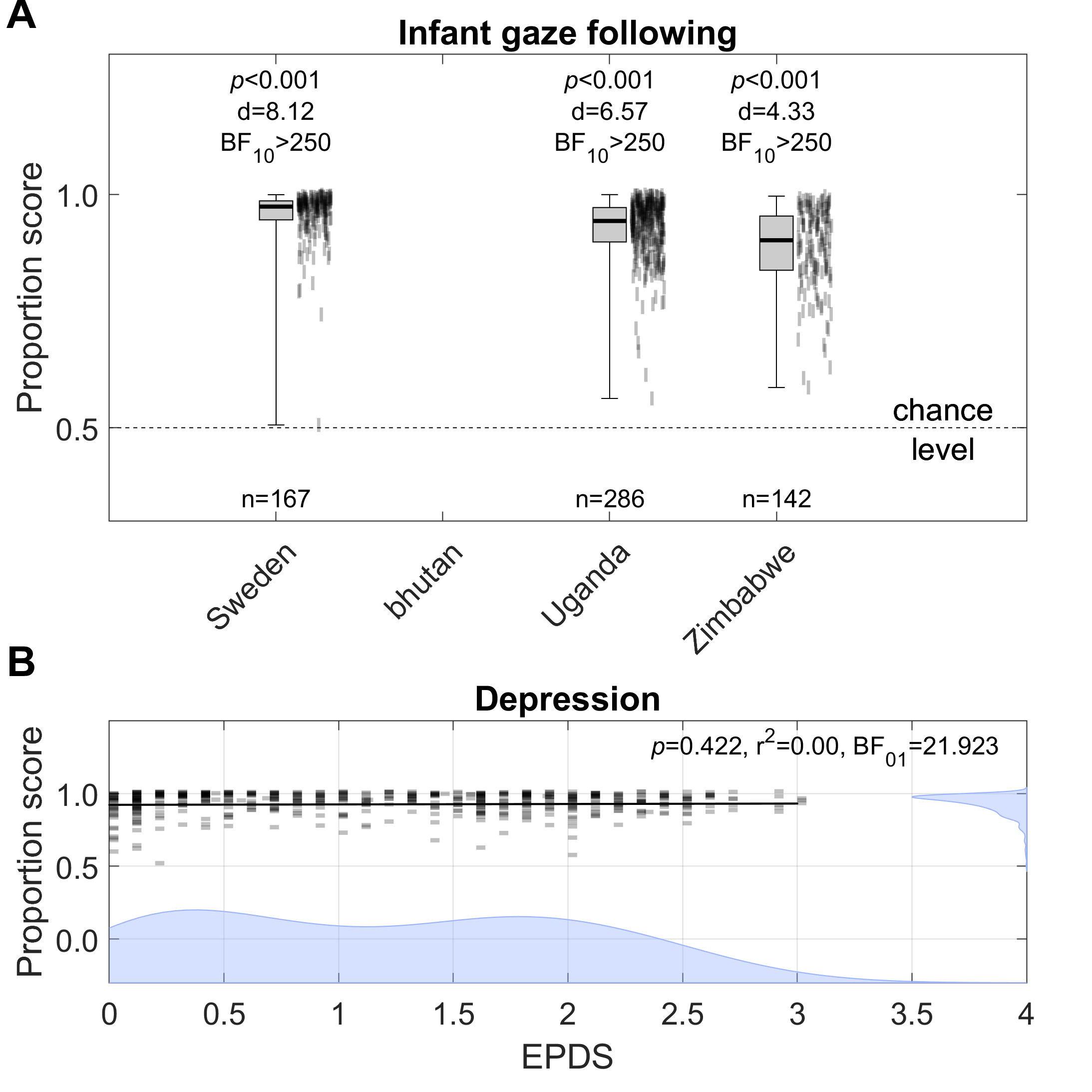

Supplement: sj-png-6-pss-10.1177_09567976251331042 – Supplemental material for Infant Gaze Following Is Stable Across Markedly Different Cultures and Resilient to Family Adversities Associated With War and Climate Change [file sj-png-6-pss-10.1177_09567976251331042.png]

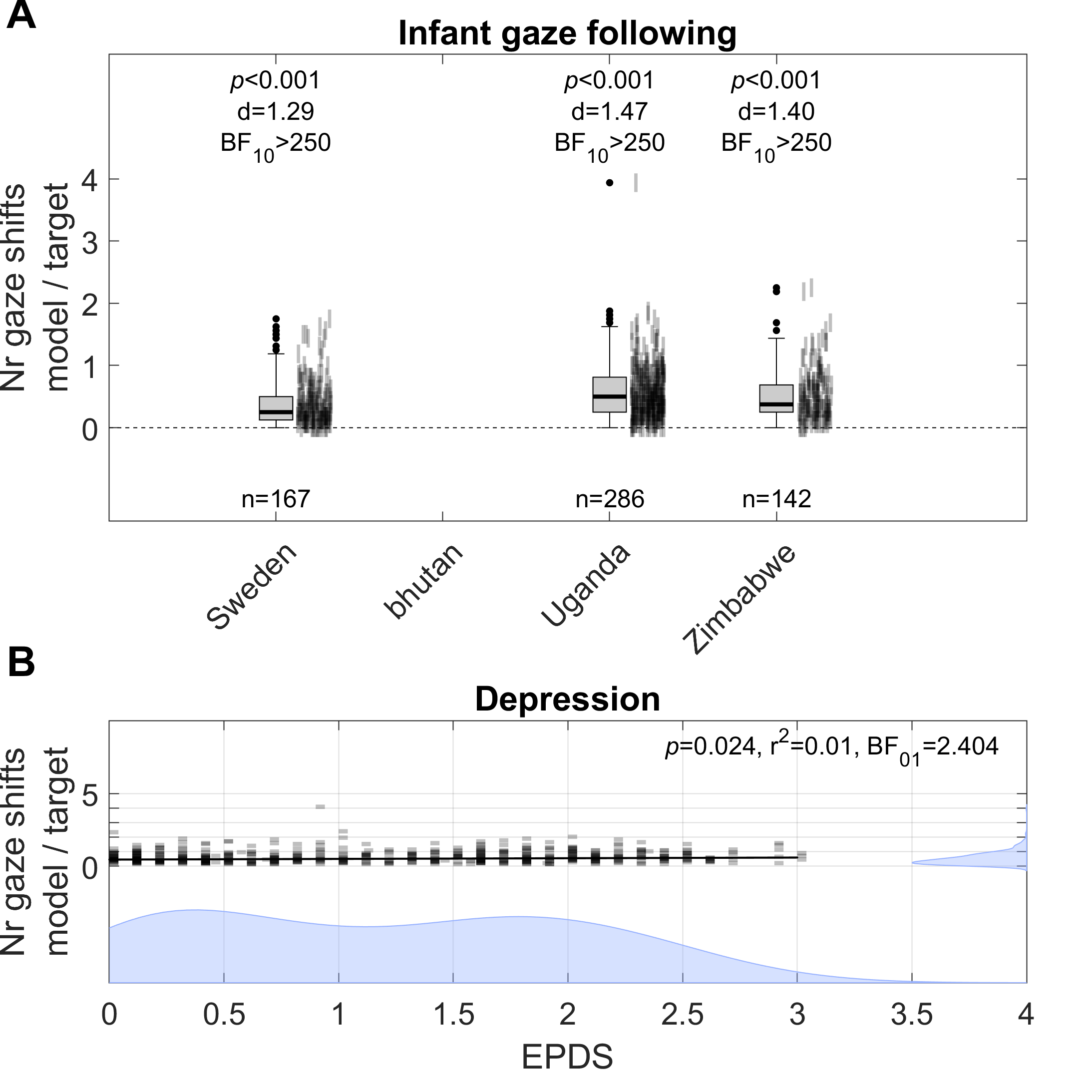

Supplement: sj-png-7-pss-10.1177_09567976251331042 – Supplemental material for Infant Gaze Following Is Stable Across Markedly Different Cultures and Resilient to Family Adversities Associated With War and Climate Change [file sj-png-7-pss-10.1177_09567976251331042.png]

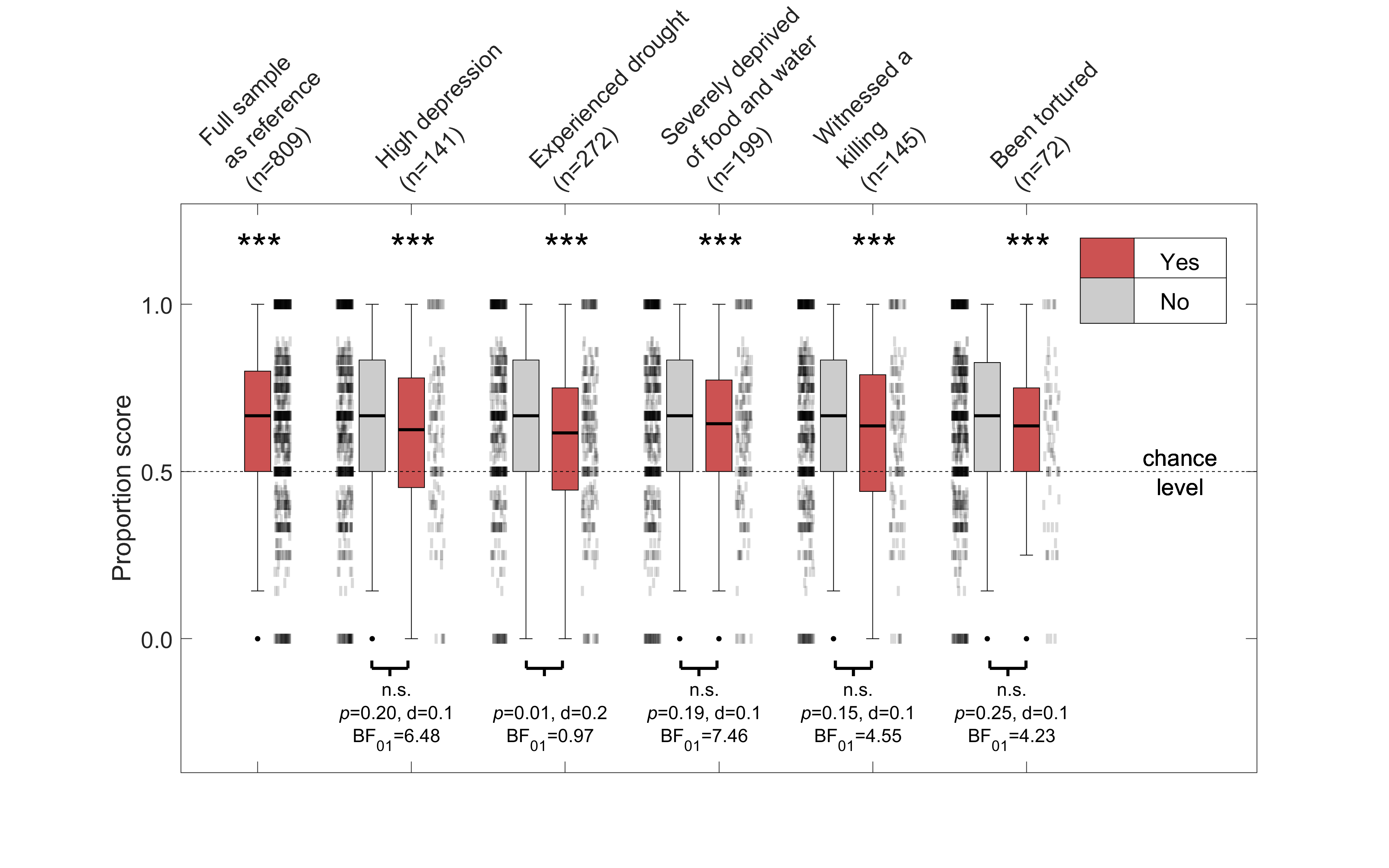

Supplement: sj-png-8-pss-10.1177_09567976251331042 – Supplemental material for Infant Gaze Following Is Stable Across Markedly Different Cultures and Resilient to Family Adversities Associated With War and Climate Change [file sj-png-8-pss-10.1177_09567976251331042.png]

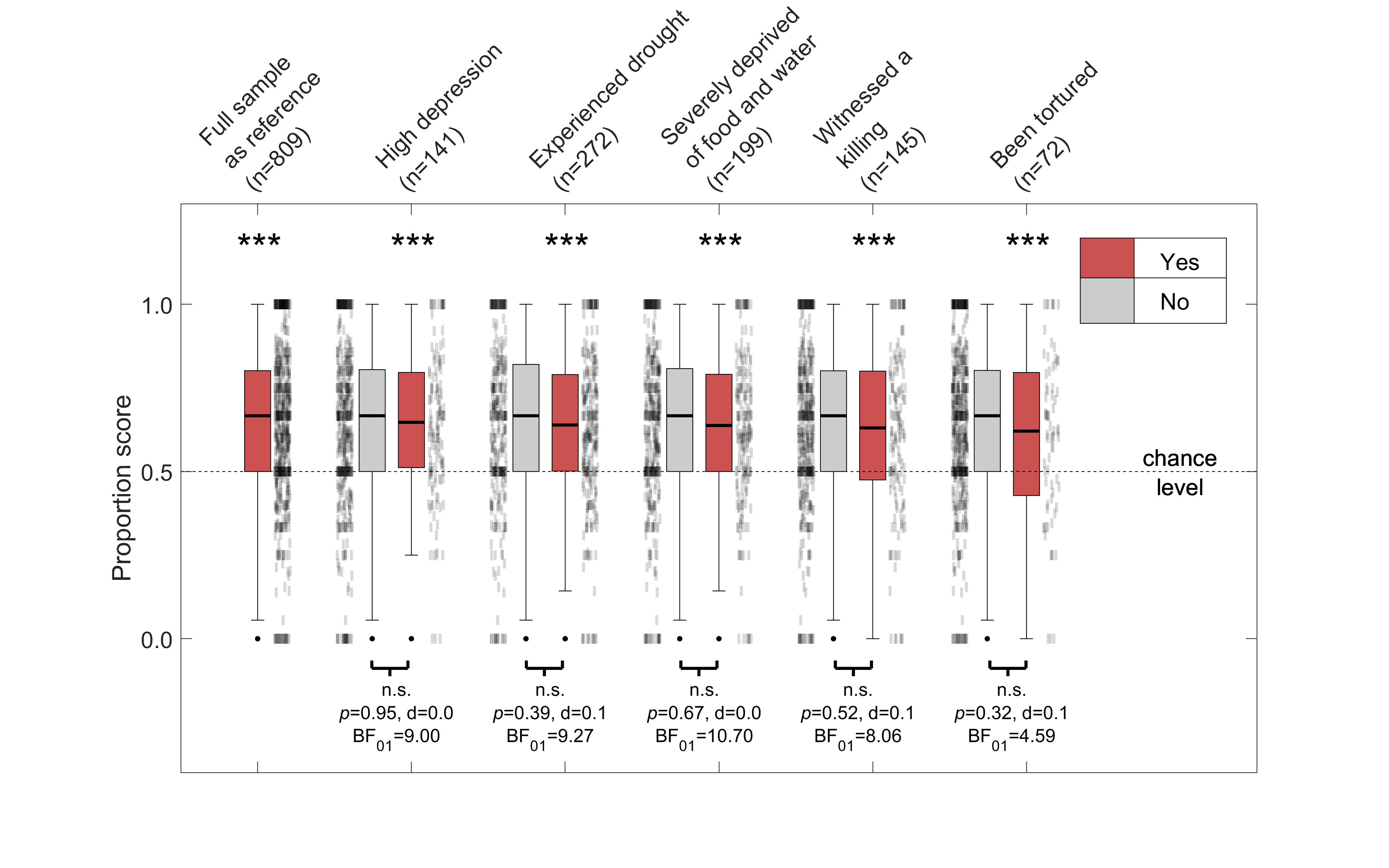

Supplement: sj-png-9-pss-10.1177_09567976251331042 – Supplemental material for Infant Gaze Following Is Stable Across Markedly Different Cultures and Resilient to Family Adversities Associated With War and Climate Change [file sj-png-9-pss-10.1177_09567976251331042.png]

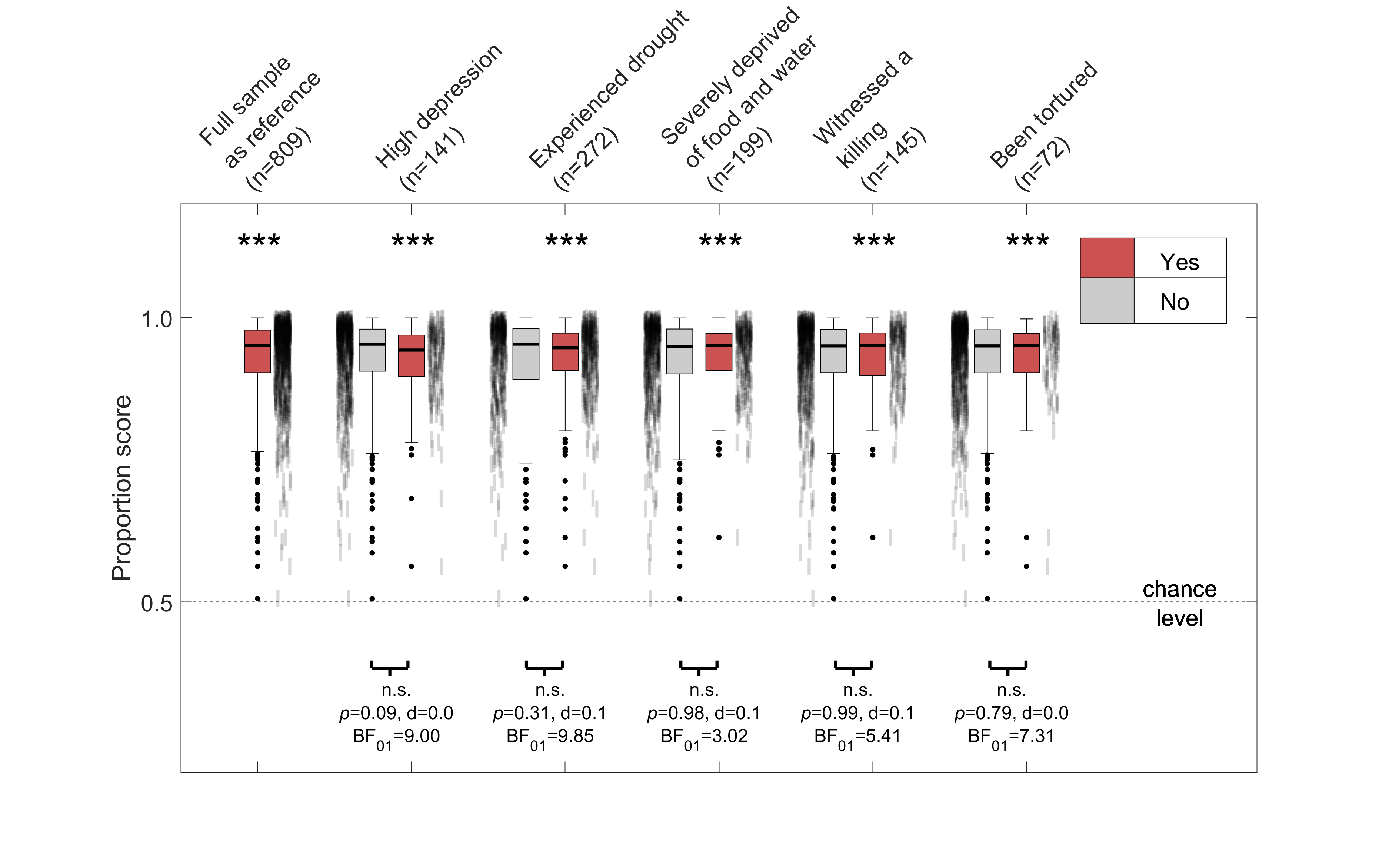

Supplement: sj-png-10-pss-10.1177_09567976251331042 – Supplemental material for Infant Gaze Following Is Stable Across Markedly Different Cultures and Resilient to Family Adversities Associated With War and Climate Change [file sj-png-10-pss-10.1177_09567976251331042.png]

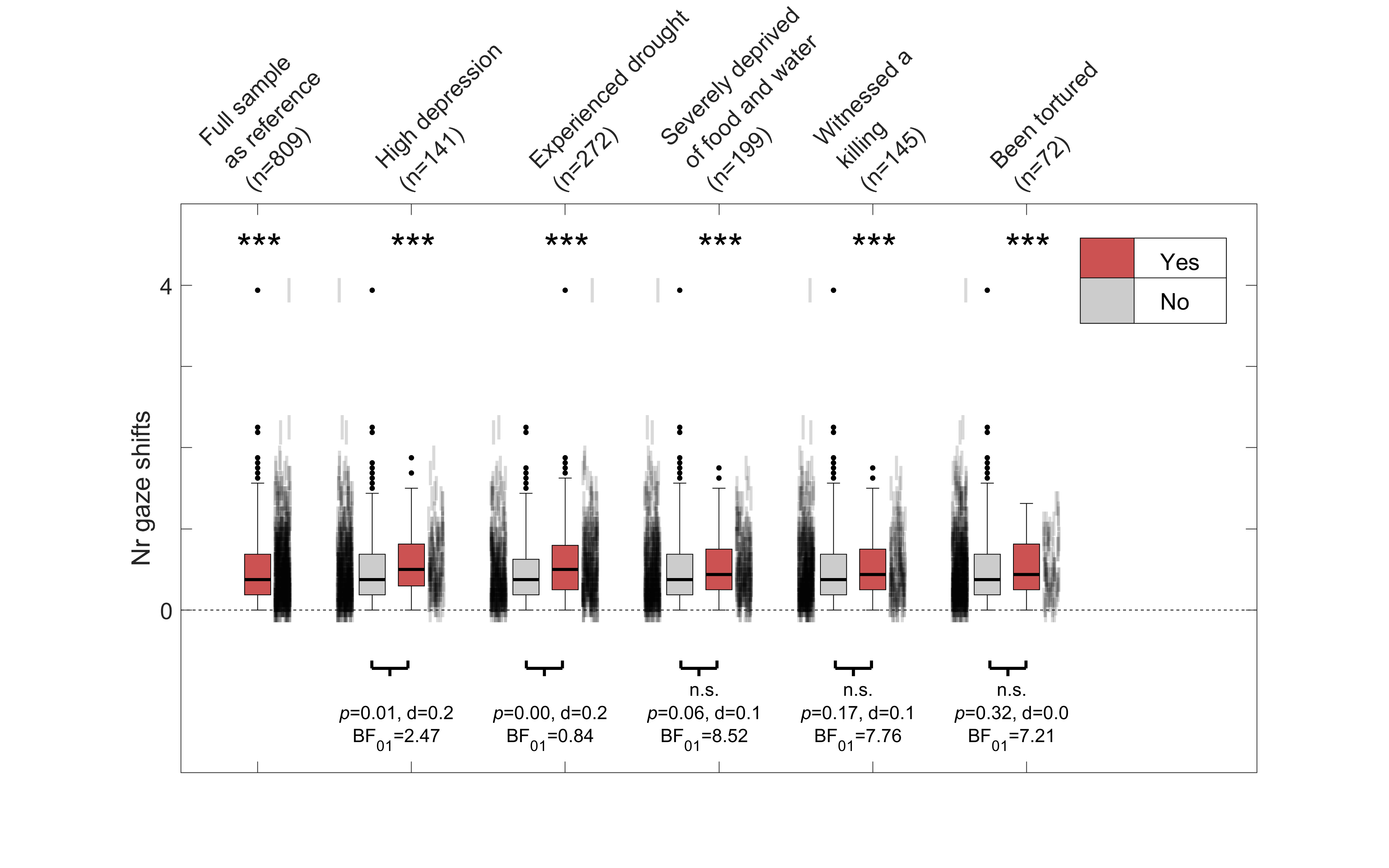

Supplement: sj-png-11-pss-10.1177_09567976251331042 – Supplemental material for Infant Gaze Following Is Stable Across Markedly Different Cultures and Resilient to Family Adversities Associated With War and Climate Change [file sj-png-11-pss-10.1177_09567976251331042.png]

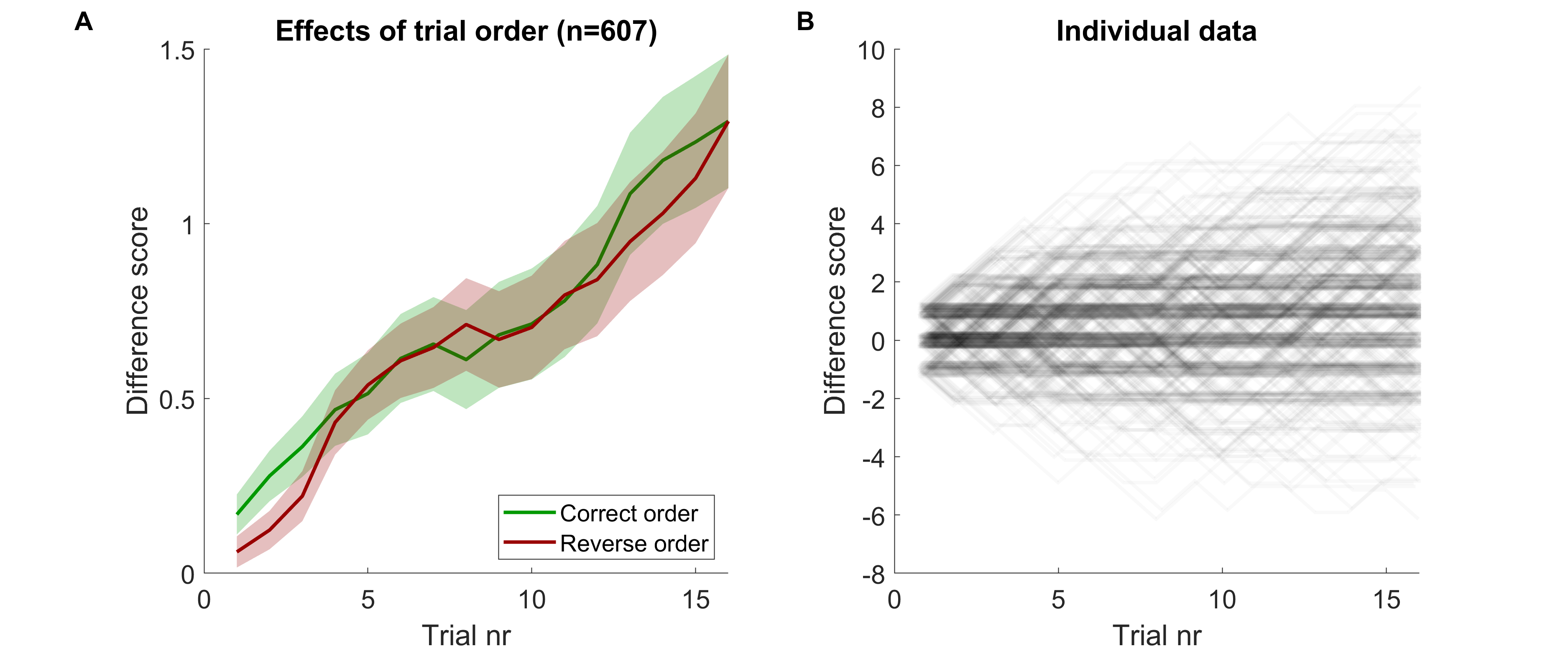

Supplement: sj-png-12-pss-10.1177_09567976251331042 – Supplemental material for Infant Gaze Following Is Stable Across Markedly Different Cultures and Resilient to Family Adversities Associated With War and Climate Change [file sj-png-12-pss-10.1177_09567976251331042.png]
